# Describing the Process of Adopting Nutrition and Fitness Apps: Behavior Stage Model Approach

**DOI:** 10.2196/mhealth.8261

**Published:** 2018-03-13

**Authors:** Laura M König, Gudrun Sproesser, Harald T Schupp, Britta Renner

**Affiliations:** ^1^ University of Konstanz Konstanz Germany

**Keywords:** mHealth, eating, physical activity, exercise, smartphone, mobile applications, health promotion

## Abstract

**Background:**

Although mobile technologies such as smartphone apps are promising means for motivating people to adopt a healthier lifestyle (mHealth apps), previous studies have shown low adoption and continued use rates. Developing the means to address this issue requires further understanding of mHealth app nonusers and adoption processes. This study utilized a stage model approach based on the Precaution Adoption Process Model (PAPM), which proposes that people pass through qualitatively different motivational stages when adopting a behavior.

**Objective:**

To establish a better understanding of between-stage transitions during app adoption, this study aimed to investigate the adoption process of nutrition and fitness app usage, and the sociodemographic and behavioral characteristics and decision-making style preferences of people at different adoption stages.

**Methods:**

Participants (N=1236) were recruited onsite within the cohort study Konstanz Life Study. Use of mobile devices and nutrition and fitness apps, 5 behavior adoption stages of using nutrition and fitness apps, preference for intuition and deliberation in eating decision-making (E-PID), healthy eating style, sociodemographic variables, and body mass index (BMI) were assessed.

**Results:**

Analysis of the 5 behavior adoption stages showed that stage 1 (“unengaged”) was the most prevalent motivational stage for both nutrition and fitness app use, with half of the participants stating that they had never thought about using a nutrition app (52.41%, 533/1017), whereas less than one-third stated they had never thought about using a fitness app (29.25%, 301/1029). “Unengaged” nonusers (stage 1) showed a higher preference for an intuitive decision-making style when making eating decisions, whereas those who were already “acting” (stage 4) showed a greater preference for a deliberative decision-making style (*F*_4,1012_=21.83, *P*<.001). Furthermore, participants differed widely in their readiness to adopt nutrition and fitness apps, ranging from having “decided to” but not yet begun to act (stage 2; nutrition: 6.88%, 70/1017; fitness: 9.23%, 95/1029) to being “disengaged” following previous adoption (stage 5; nutrition: 13.77%, 140/1017; fitness: 15.06%, 155/1029).

**Conclusions:**

Using a behavior stage model approach to describe the process of adopting nutrition and fitness apps revealed motivational stage differences between nonusers (being “unengaged,” having “decided not to act,” having “decided to act,” and being “disengaged”), which might contribute to a better understanding of the process of adopting mHealth apps and thus inform the future development of digital interventions. This study highlights that new user groups might be better reached by apps designed to address a more intuitive decision-making style.

## Introduction

In recent years, services supporting medical and public health practices via mobile technology (mHealth) [1] such as smartphone apps have become increasingly popular. More than 70,000 mHealth apps are currently available for download on Android and iOS smartphones [2], and more apps are released every year [3]. The proportion of smartphone owners currently using an mHealth app ranges between 36% [4] and 58% [5] in the United States and between 11% [6] and 21% [7] in Germany, where this study was conducted. Although mHealth apps have the potential to deliver effective interventions [8-12] and cut health care costs [13,14], for example, because medical interventions can be delivered remotely instead of in person, a large proportion of the population does not actively use mHealth apps [15]. The European Union therefore set a goal to make Web-based health promotion, including mHealth apps, more effective, user-friendly, and widely acceptable [16,17].

A first step to attaining this goal is to identify who is currently using mHealth apps and who is not. Usually, studies divide the participants into a “user group,” comprising participants who currently use an mHealth app (eg, [6]) or have one installed (eg, [4,18]), and a “nonuser group,” which typically lacks further specification. Few studies have described mHealth app users and nonusers using sociodemographic and health-related characteristics or assessed further information about nonusers, such as discontinued mHealth app use (eg, [5,19]) or interest in mHealth app use (eg, [20,21]). Compared with nonusers, mHealth app users tend to have more education and are younger [18]. All genders use mHealth apps equally often [4-7,22,23]. Regarding health-related parameters, such as current health status or body mass index (BMI), research yielded mixed results. Although some suggest that mHealth app users tend to be healthier and less likely to be overweight [24,25], others report more comorbidities and a higher BMI for users [4,7].

However, more than a basic understanding of the core sociodemographic characteristics of users and nonusers is needed to increase mHealth app adoption rates. That is, we require a better understanding of the motivational processes underlying the decision making for adopting mHealth apps. In health behavior research, stage theories of behavior change [26-29] suggest that people can be differentiated according to the levels of awareness of and motivation to adopt a healthier lifestyle, such as quit smoking [30], become more physically active [31], change dietary behaviors [32-35], or to take preventive action such as increasing calcium intake to prevent osteoporosis [33]. Specifically, stage models such as the Transtheoretical Model of Health Behavior Change (TTM) [36,37], the Health Action Process Approach (HAPA) [26,38], or the Precaution Adoption Process Model (PAPM) [39-41] assume that people pass through qualitatively different motivational stages when adopting a behavior (see [27,42] for an overview). For example, the PAPM claims that people pass through 7 distinct stages of decision making for health behavior, including being “unaware,” “becoming engaged,” “starting to make a decision,” “decided to act,” “decided not to act,” “acting,” and finally “maintaining” (or “disengaging”) from the behavior [39,43]. Importantly, the PAPM introduced differentiation between people who have “decided not to act” and people who are yet undecided. People who have already formed an opinion about an issue might be more difficult to persuade than people who did not yet form an opinion, and therefore might require different intervention approaches [40,41]. Furthermore, in the PAPM, stages are defined by psychological characteristics instead of external factors such as time, as in the TTM [28,41], which has been criticized as being a rather arbitrary criterion [44]. Using stage models to describe a person’s position in the behavioral adoption process has been shown to improve recruitment, retention, and progress in the behavior change process [36,37] by providing information about barriers of change for individual stages as well as methods to facilitate stage transitions [36,40,43]. Drawing on the stage model conception from health psychology research and especially the PAPM, we used a stage model approach to assess 5 different stages in the adoption process of mHealth apps. In particular, the 5 different stages include those who have never thought about using mHealth apps (“unengaged”), intend to use mHealth apps in the future (“decided to act”), have decided against using mHealth apps (“decided not to act”), are currently using mHealth apps (“acting”), and have ceased to use mHealth apps (“disengaged”). The later stage was added based on a previous adaptation of the PAPM [45], because comparing “disengaged” nonusers to other groups, especially “acting” users, provides valuable information about when and why mHealth app use is maintained or discontinued [19]. Thus, the present stage model also includes the perspective of models of engagement with digital behavior change interventions that focus on preventing the transition from the “acting” stage to disengagement.

When stages of mHealth app adoption have been identified, a second and important step is to characterize the people at each stage to identify potential transition barriers [43]. Characterizing groups at each stage is important to both tailoring and improving the services according to users’ needs and preferences and thereby enhancing user engagement and promoting the use of mHealth apps to new user groups [46-48]. The extent of mHealth app use, for example, seems to covary with health consciousness, health information orientation, and eHealth literacy [49]. These results suggest that mHealth apps are more likely to be adopted by people who are conscious about their health. Research in health screening decision-making furthermore showed that decision-making styles affect information processing. Specifically, people with a rational decision-making style engaged more with intervention materials such as leaflets than those with an intuitive decision-making style [50]. As mHealth apps that are currently available predominantly focus on self-regulatory strategies such as self-monitoring, providing instruction or feedback, and goal setting [51-53], using mHealth apps might necessitate self-regulatory competencies such as a deliberative decision-making style. Similarly, previous research suggests that self-regulatory constructs that support goal-directed, intentional behaviors (eg, self-efficacy, attitudes) may act as transition barriers in the PAPM [34]. Consequently, people who use a deliberate style when making health-related decisions, such as preferring to rely on health recommendations, may be more likely to adopt mHealth apps. A preference for deliberation might help to exert the self-control needed to perform the behavior. Conversely, people who prefer an intuitive decision-making style, that is, relying on affect and heuristics [54,55], might be less likely to adopt mHealth apps as such apps tend to stand in stark contrast to their preferred decision-making strategies. Accordingly, decision-making style preferences might systematically relate to stages in the adoption process.

Although mHealth apps have different functionalities, the majority of available apps are targeted at lifestyle and well-being, with the majority being designed to monitor eating behavior and physical activity [56,57]. Previous research, however, predominantly focused on investigating use and nonuse of mHealth apps in general, instead of investigating the use or nonuse of different categories separately (eg, [4,5,7]). However, the use of mHealth apps that target different behavioral domains, for example, eating or physical activity, might be correlated with different sociodemographic, behavioral, or psychological characteristics. For instance, women are more strongly preoccupied with eating [58]; thus, one might expect that women are more interested in nutrition apps than men. Therefore, this study focused on nutrition apps, but also included fitness apps to examine whether the results are behavior-specific or generalize across behavioral domains.

The aims of this study are twofold. First, it aimed to investigate different stages in the adoption process of nutrition and fitness apps by utilizing a newly developed stage model based on the PAPM. Second, building upon and extending previous research, the study aimed to investigate sociodemographic, behavioral, and psychological characteristics of people at the different adoption stages for nutrition apps to inform a better understanding of stage transitions. Specifically, we assumed that an intuitive decision-making style might act as a transition barrier and thus is more pronounced in participants who are not “acting.”

## Methods

### Design and Procedure

Data were collected as part of the Konstanz Life Study, an ongoing longitudinal cohort study that was launched in spring 2012 with 1321 participants (for more details, see [59-63]). The overarching aim of the study is to investigate psychological influences on eating behavior, physical activity, and health within the general population across time [59]. The study was part of the SMARTACT research project funded by the German Federal Ministry of Education and Research. Further points of measurement, 2, 3, and 4, took place in autumn 2012, spring 2013, and spring 2016, respectively. For each point of measurement, participants were recruited via flyers, posters, and newspaper articles. Additionally, participants of the preceding points of measurement were reinvited via email and phone calls. People aged 18 years and older without acute infectious diseases were eligible for participation. The measurements included the collection of fasting blood samples, questionnaires, as well as a standardized check-up including anthropometric measures and cognitive and physical fitness tests. As compensation for participation, participants received feedback about their objective health status referenced to the current norms. This paper presents questionnaire and anthropometric data collected in the fourth point of measurement (spring 2016).

### Ethics

For data processing and security, a register of processing operations was developed in cooperation with and approved by ZENDAS in 2012 and reviewed in 2016 (Zentrale Datenschutzstelle der Baden-Württembergischen Universitäten/ Center for Data Protection of the Universities in Baden-Württemberg) and reviewed by the Landesdatenschutz- Beauftragte, Baden-Württemberg (Commissioner for Data Protection in Baden-Württemberg). All participants gave written informed consent before participation. The study adhered to the guidelines of the German Psychological Society (Deutsche Gesellschaft für Psychologie) and the Declaration of Helsinki, and was conducted in compliance with relevant laws and institutional guidelines. The study protocol was approved by the University of Konstanz ethics committee.

### Sample

**Figure 1:**
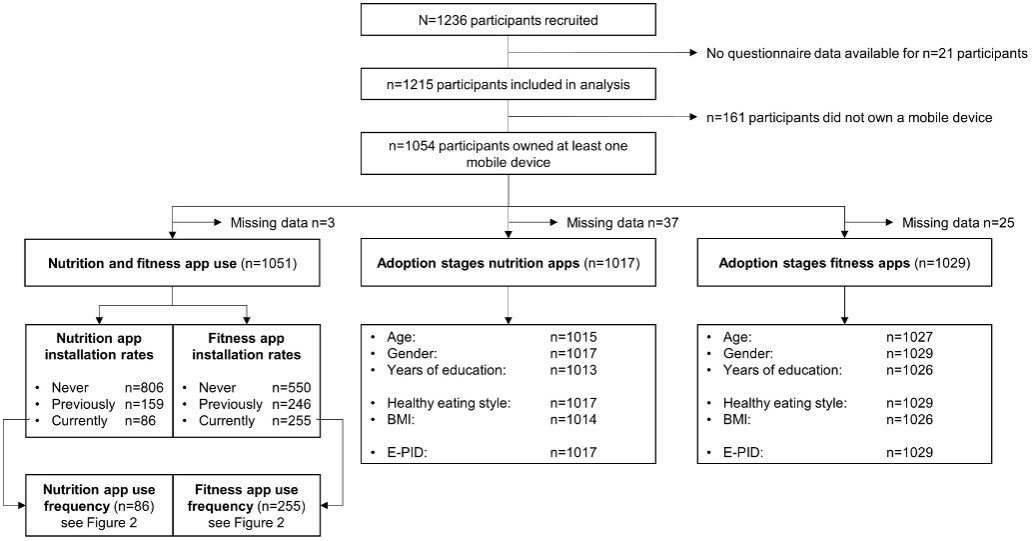
Flowchart of the study sample.

In total, 1236 participants were recruited for the fourth wave. For 21 participants, no questionnaire data were obtained, reducing the sample analyzed to 1215 (for a detailed overview, see Figure 1). The sample had a mean age of 41.11 years (SD 17.56) and 64.44% (783/1215) were female. BMI ranged from 16.77 to 42.45 kg/m² (mean 24.21 [SD 3.63]). The majority of participants had a university entrance diploma (71.26%, 858/1204), and 53.16% (640/1204) had a university degree. Compared with the German population, the sample consisted of 13.7% more females, was 3.19 years younger, and had a lower BMI by 1.69 points [64,65]. Furthermore, the present sample was better educated than the general German population, in that 29.5% have a university entrance diploma and 16.3% have a university degree [66].

### Measures

#### Mobile Device Ownership and Nutrition and Fitness App Use

Participants were asked to indicate whether they owned a smartphone or tablet, (1) yes; (2) no. If the participants owned a mobile device, they were subsequently asked to indicate whether they had ever installed an app to monitor their physical activity (fitness app) or their eating behavior (nutrition app) on a 4-point Likert scale ranging from (1) never to (4) currently. If they indicated that they currently had a fitness or nutrition app installed on their mobile device, they were further asked to indicate the frequency of use on a 5-point Likert scale ranging from (1) once a month or less to (5) at least once a day.

#### Stage Model for the Adoption Process of mHealth Apps (Nutrition and Fitness)

For this study, in accordance with the PAPM [40] and an adaptation of the PAPM by Renner and Hahn [45] (see also Multimedia Appendix 1), we defined each participant’s stage in the adoption process based on their response to 5 different statements representing the different stages. Participants were asked to choose the one statement they would agree with most regarding the usage of an mHealth app for physical activity or food intake. Participants were categorized using the following 5 behavior adoption stages: (stage 1) being “unengaged” (“I have never thought about using an app for that [nutrition/fitness]”), (stage 2) “decided to act” (“I have thought about using an app for that [nutrition/fitness], but so far I did not do it”), (stage 3) “decided not to act” (“I have thought about using an app for that [nutrition/fitness], but it is not necessary for me to do it”), (stage 4) “acting” (“I am currently using an app for that [nutrition/fitness] and intend to continue to use it”), and (stage 5) being “disengaged” (“I have used an app for that [nutrition/fitness], but I do not use it anymore”). Stages 1-3 and 5 encompass nonusers, whereas stage 4 includes current users.

#### Preference for Intuition and Deliberation in Eating Decision-Making

A 7-item scale was used to measure the habitual preference for intuition and deliberation in eating decision-making (E-PID; unpublished data [67]; see also Multimedia Appendix 1). The E-PID scale, consisting of 2 subscales, was developed based on the inventory for preference for intuition and deliberation by Betsch [54]. Participants answered each item on a 5-point Likert scale from (1) I do not agree to (5) I agree. A confirmatory factor analysis was conducted using a latent structural equation model in MPlus to test the hypothesized two-factor structure. The comparative fit index (CFI=.988), root mean square error of approximation (RMSEA=.048, 90% CI 0.034-0.062), and the standard root mean square residual (SRMR=.024) indicated a good model fit [68]. All items showed statistically significant factor loadings (*P* s<.001), indicating convergent validity. The first factor “preference for intuition” (E-PI) consisted of 3 items (eg, “When deciding what to eat, I rely on my gut feeling.”; mean 3.34 [SD 0.83], alpha=.78) that describe decision making based on feelings or affect (cf Betsch [54]). The second factor “preference for deliberation” (E-PD) consisted of 4 items (eg, “I prefer making plans about my eating behavior instead of leaving it to chance.”; mean 3.19 [SD 0.95], alpha=.84) that describe decision making based on deliberation and planning.

#### Healthy Eating Style

Healthy eating style was measured with 16 items assessing general food consumption patterns (eg, “I do not eat fast food,” “I only eat foods containing little salt,” “If I eat sweets or cakes, I only eat little,” and “I eat a lot of fruit and fresh vegetables”) using a 7-point Likert scale from (1) strongly disagree to (7) strongly agree (cf, Renner et al [69], Leppin [70]). To investigate the factor structure, an exploratory factor analysis was conducted using a principal component analysis and promax rotation. Global diagnostic indicators showed adequate factorability of the correlation matrix, with Kaiser-Meyer-Olkin=.81 and a significant Bartlett test of sphericity (χ^2^_120_=3106.1, *P<*.001). Both eigenvalues on the scree-plot as well as the MAP test [71] suggested a one-factor solution. A total of 4 items were excluded because they loaded less than λ=.30 on the factor, yielding a 12-item scale that accounted for 29.39% of the variance. Items were aggregated, and a higher score represents a healthier eating style (mean 4.34 [SD 0.90], alpha=.77).

#### Body Mass Index

BMI was calculated using the height and weight measurements taken by trained research staff following a standardized procedure. Participants wore light indoor clothing and were asked to take off their shoes. Height was measured using a wall-mounted stadiometer, and weight was measured using a digital scale (Omron Body Composition Monitor, BF511).

#### Sociodemographic Variables

Participants’ age and gender were assessed. Additionally, participants’ level of education was assessed and converted into years of education.

Means and standard deviations are listed in Table 1 for nutrition apps and in Multimedia Appendix 2 for fitness apps.

**Table 1:** Descriptive statistics of correlates of nutrition app adoption.

Stages of behavioral adoption	Gender^a^, n (standardized adjusted residuals)			Age, mean (SD)	Years of education, mean (SD)	BMI^b^, mean (SD)	Healthy eating style, mean (SD)
	Female	Male	*P* value				
Stage 1 “unengaged”	312 (−3.25)	221 (3.25)	.001	41.33 (15.88)	16.18 (2.33)	24.01 (3.24)	4.29 (0.92)
Stage 2 “decided to act”	44 (−0.07)	26 (0.07)	.94	37.33 (16.28)	15.06 (2.54)	24.86 (4.17)	4.08 (0.94)
Stage 3 “decided not to act”	126 (0.77)	66 (−0.77)	.44	35.15 (15.35)	15.89 (2.43)	23.63 (3.32)	4.26 (0.79)
Stage 4 “acting”	58 (1.47)	24 (−1.74)	.14	32.93 (14.14)	15.10 (2.44)	24.44 (3.49)	4.50 (0.84)
Stage 5 “disengaged”	103 (2.73)	37 (−2.73)	.006	32.16 (12.91)	15.69 (2.28)	24.24 (4.26)	4.29 (0.86)

^a^For gender, the number of participants in the cell and the standardized adjusted residuals (in brackets) are displayed. Due to multiple comparisons, the significance level was adjusted to alpha=.005.

^b^BMI: body mass index.

### Statistical Analysis

Analyses were performed using IBM SPSS Statistics (Version 23). Missing values were 0.00% (0/1215) for gender, 0.08% (1/1215) for healthy eating style and E-PID, 0.16% (2/1215) for age, 0.25% (3/1215) for BMI, 1.4% (17/1215) for years of education and ownership of mobile devices, and 6.09% (74/1215) for fitness and 7.74% (94/1215) for nutrition app adoption stages. Participants with missing data on a variable relevant to an analysis were excluded for that specific analysis only. Descriptive statistics are reported for the full dataset (N=1215). All analyses on differences between nutrition and fitness app use stages were conducted using a subsample that had indicated owning at least one mobile device (N=1054). To investigate differences between nutrition and fitness app use stages by age, years of education, BMI, and healthy eating style, one-way analyses of variance (ANOVA) were conducted. Post hoc analyses were conducted using Bonferroni correction. Levene tests were conducted to test for the precondition of homogeneity of variances. This precondition was not met for analyzing differences in age (*F*_4,1010_=7.84, *P*<.001) or BMI for nutrition app adoption stages (*F*_4,1009_=3.27, *P*=.011) or for age differences between fitness app adoption stages (*F*_4,1022_=8.00, *P*<.001). To analyze these relationships, Welch tests and Games-Howell post hoc tests were conducted. Gender differences were examined using chi-square tests. Post hoc tests were performed using standardized residuals and Bonferroni correction [72]. Adoption stage differences in preference for intuition and deliberation were analyzed using mixed ANOVAs, with Stages of Behavioral Adoption as a between-subjects factor and E-PID as a within-subjects factor. Significant results were followed up by simple effects (cf, Page et al [73]). For these comparisons, the alpha level was adjusted to .001 to account for multiple comparisons.

## Results

### Mobile Devices and Nutrition and Fitness App Use

Of the total sample, 84.95% (1010/1189) of participants indicated owning a smartphone, and 40.89% (480/1174) owned a tablet. Taken together, 1054 (87.98%) of the study population owned at least 1 mobile device that allowed them to use apps.

Installation rates of nutrition and fitness apps were further investigated in the subsample that owned at least 1 mobile device (see Figure 1). Of all the participants, 76.69% (806/1051) indicated that they never had installed a nutrition app, 15.13% (159/1051) had previously installed one, and 8.18% (86/1051) reported having one currently installed on their mobile device. For fitness apps, 52.33% (550/1051) reported never having had a fitness app installed, 23.41% (246/1051) had had one installed previously, and 24.26% (255/1051) currently had one installed on their smartphone or tablet.

**Figure 2:**
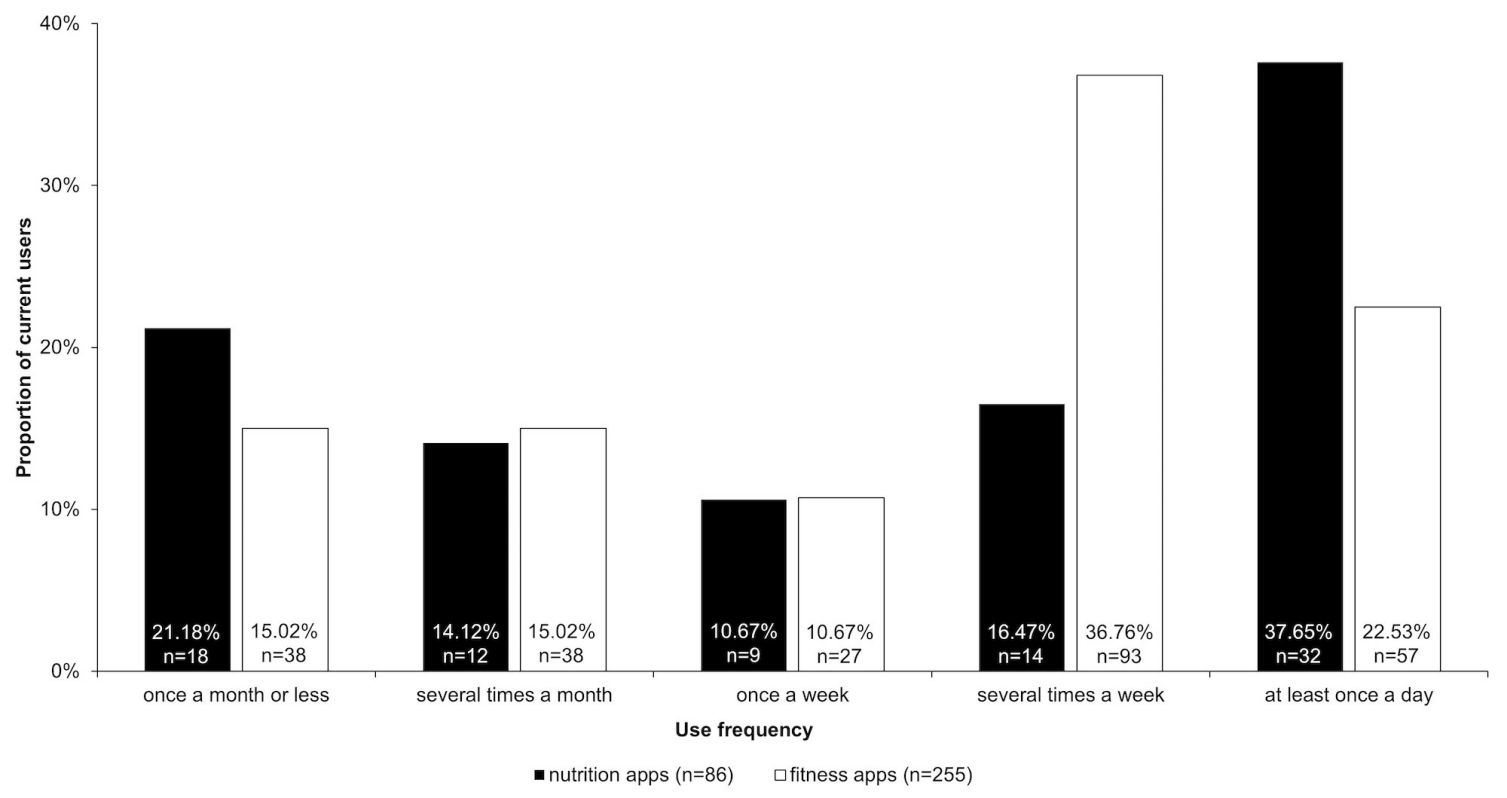
Frequency of use of nutrition (n=86) and fitness apps (n=255).

In a next step, frequency of use was investigated in those participants who had indicated having a currently installed a nutrition (n=86) or fitness app (n=255) on their mobile device (for a summary, see Figure 2). For nutrition apps, most participants indicated using the app at least once a day (37.65%, 32/86), whereas for fitness apps, the largest proportion of participants indicated that they used a fitness app several times a week (36.7%, 93/255).

### Stages of Behavioral Adoption

**Figure 3:**
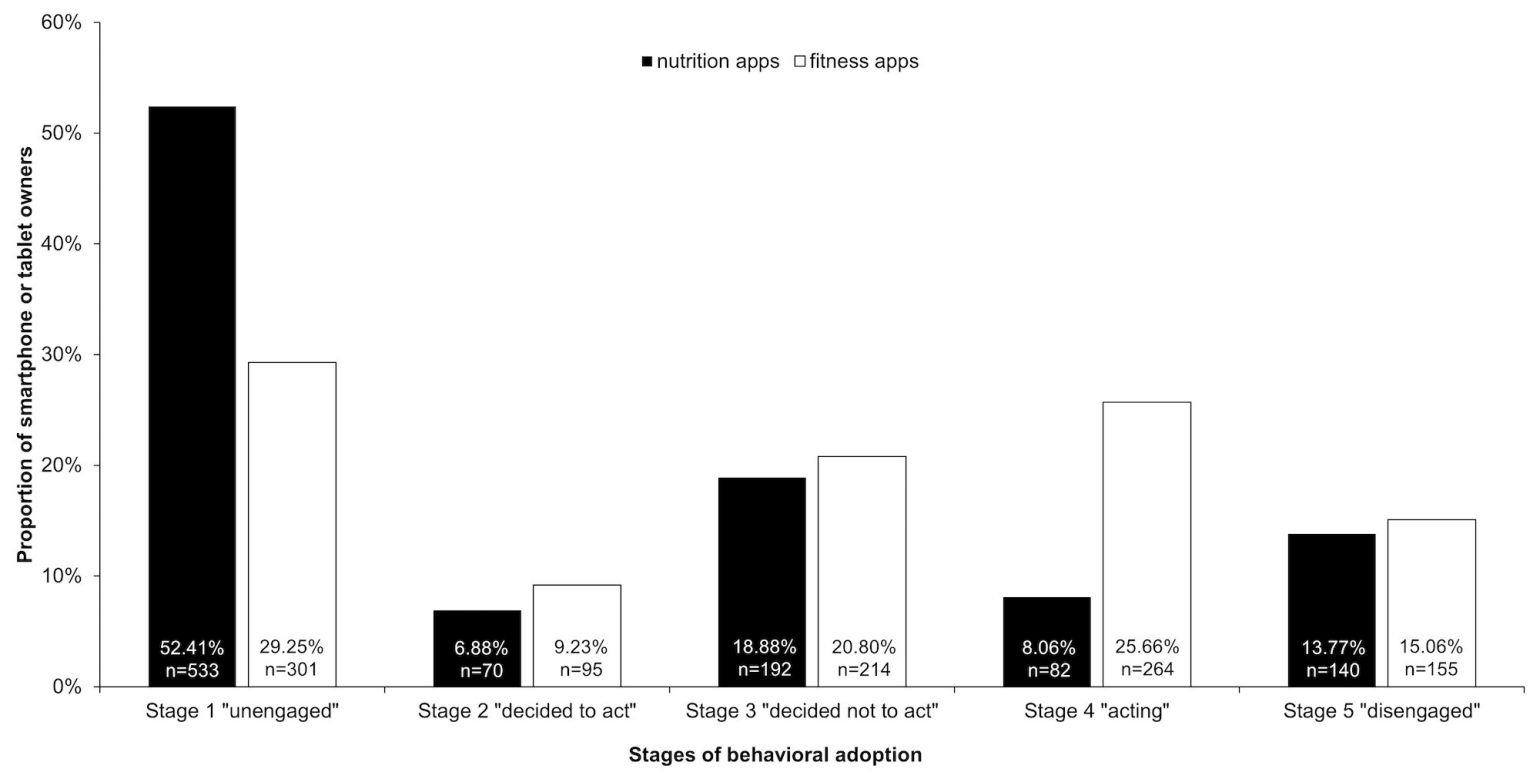
Stages of behavioral adoption of nutrition and fitness apps.

Of all the participants who owned a mobile device (see also Figure 3; means and standard deviations are listed in Table 1), 52.41% (533/1017) indicated that they had never thought about using a nutrition app and were therefore classified as “unengaged” nonusers (stage 1). Another 6.88% (70/1017) indicated that they are planning to use a nutrition app in the future and were thus categorized as “decided to act” nonusers (stage 2), and 18.88% (192/1017) were classified as “decided not to act” nonusers (stage 3) as they indicated having decided against using a nutrition app. Moreover, 8.06% (82/1017) indicated that they were currently using a nutrition app and categorized as “acting” users (stage 4), and 13.77% (140/1017) reported having previously used a nutrition app and were categorized as “disengaged” nonusers (stage 5).

In relation to the 5 stages of fitness app adoption, 29.25% (301/1029) of the participants who owned a mobile device were categorized as “unengaged” (stage 1), 9.23% (95/1029) as “decided to act” (stage 2), 20.80% (214/1029) as “decided not to act” (stage 3), 25.66% (264/1029) as “acting” (stage 4), and 15.06% (155/1029) as “disengaged” (stage 5) (see also Figure 3).

**Figure 4:**
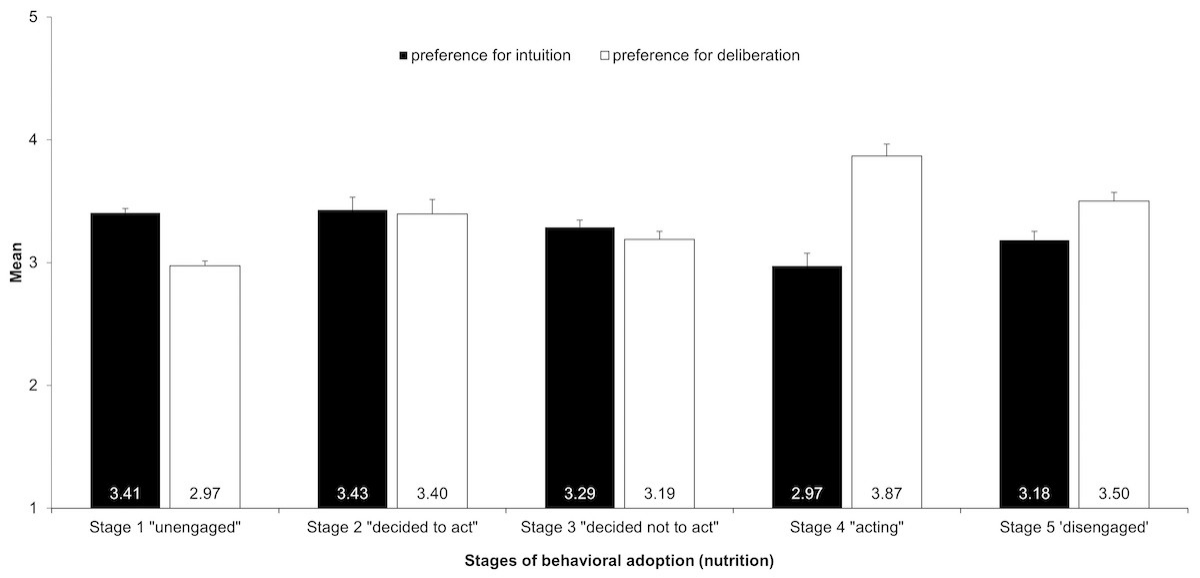
Differences in preference for intuition and deliberation between stages of behavioral adoption of nutrition apps.

### Sociodemographic Correlates

Significant age differences between the 5 stages of behavioral adoption of nutrition apps emerged (*F*_4,252.00_=16.85, *P*<.001, ω^2^=.06), with the participants in stage 1 (“unengaged”) (mean 41.33 [SD 15.88]) being older than the participants in stage 2 (“decided to act”) (mean 37.33 [SD 16.28], *P*<.001), stage 4 (“acting”) (mean 32.93 [SD 14.14], *P*<.001), and stage 5 (“disengaged”) (mean 32.16 *,* [SD 12.91], *P*<.001). Furthermore, a significant association between stages of behavioral adoption of nutrition apps and gender emerged (χ^2^_4_=14.9, *P*=.007, Cramer V=.12). Men were more often in stage 1 (“being unaware”) than women. Moreover, significant stage differences were found for years of education (*F*_4,1008_=6.65, *P*<.001, partial η^2^=.03). Post hoc tests revealed that participants in stage 1 (“unengaged”) (mean 16.18 [SD 2.33]) were better educated than participants in stage 2 (“decided to act”) (mean 15.06 [SD 2.54], *P*=.002) and stage 4 (“acting”) (mean 15.10 [SD 2.44], *P*=.001).

Further analysis of the differences between the stages of fitness app adoption showed similar age differences as for nutrition app adoption (*F*_4,398.29_=22.38, *P*<.001, ω^2^=.08). Participants in stage 1 (“unengaged”) (mean 45.31, [SD 16.61]) were significantly older than participants in the remaining 4 stages (stage 2 “decided to act”: mean 37.14 [SD 15.64], *P*<.001; stage 3 “decided not to act”: mean 36.18 [SD 15.37], *P*<.001; stage 4 “acting”: mean 34.76 [SD 13.95], *P*<.001; and stage 5 “disengaged”: mean 33.74 [SD 13.52], *P*<.001). No significant differences were found both for gender (χ^2^_4_=8.7, *P*=.07) and years of education (*F*_4,1021_=2.16, *P*=.07).

### Behavioral Correlates

For nutrition apps, no significant differences for the 5 stages of behavioral adoption were found for both healthy eating style (*F*_4,1012_=2.10, *P*=.08) and BMI (*F*_4,240.01_=1.72, *P*=.15).

For fitness apps, analyzing stage differences in healthy eating style (*F*_4,1024_=2.92, *P*=.02, η^2^=.01) revealed a tendency for stage 1 participants (“unengaged”) to report a healthier eating style (mean 4.43 [SD 0.94]) than stage 4 participants (“acting”) (mean 4.23 [SD 0.84], *P*=.07). Regarding BMI, no significant stage differences were found (*F*_4,1021_=1.71, *P*=.15).

### Psychological Correlates: Preference for Intuition and Deliberation in Eating Decision-Making

The characteristics of the different stages of behavioral adoption of nutrition apps show that participants differed significantly in terms of their preference for a deliberative or an intuitive style when making eating-related decisions (see Figure 4; see also Table 1). Specifically, a 5 Stages of Behavioral Adoption (Nutrition) × 2 E-PID mixed ANOVA yielded significant results. Both a main effect for the between-subjects factor Stages of Behavioral Adoption (F_4,1012_=6.96, *P*<.001, partial η^2^=.03) and a main effect for the within-subjects factor E-PID (*F*_1,1012_=5.21, *P*=.02, partial η^2^=.01) emerged. Moreover, the interaction of the 2 factors was significant (*F*_4,1012_=21.69, *P*<.001, partial η^2^=.08). The interaction effect was followed up by simple effects to test differences between E-PI and E-PD at all levels of the Stages of Behavioral Adoption. Significant differences emerged between stage 1 (“unengaged”) (*F*_1,1012_=49.55, *P*<.001) and stage 4 (“acting”) (*F*_1,1012_=32.80, *P*<.001). Although stage 1 (“unengaged”) participants preferred on average a more intuitive eating decision-making style, stage 4 (“acting”) participants preferred on average a more deliberative eating decision-making style.

A 5 Stage of Behavioral Adoption (Fitness) × 2 E-PID mixed ANOVA was conducted to analyze stage differences in terms of the preference for a deliberative or intuitive style when making eating-related decisions to examine whether the stage characteristics are behavior specific or also generalize to the fitness app adoption process. The interaction between the between-subjects factor Stage of Behavioral Adoption (Fitness) and the within-subjects factor E-PID reached significance (*F*_4,1024_=6.17, *P*<.001, partial η^2^=.02). The interaction effect was followed up by simple effects, testing differences between E-PI and E-PD at all 5 stages. A significant difference emerged only for the participants in stage 1 (“unengaged”), with a higher preference for an intuitive style when making eating decisions (mean_E-PI_ 3.41 [SD_E-PI_=0.84]; mean_E-PD_ 3.00 [SD_E-PD_ 0.98]; *P*<.001).

## Discussion

### Nutrition and Fitness App Use

In this study, the adoption process of nutrition and fitness apps and associated characteristics were investigated using a stage model approach. The present data show that there is a great potential for mHealth apps, as more than 80% of the participants owned a mobile device, whereas only 8% of them were using a nutrition app and 26% were using a fitness app. In line with other studies, the results show that fitness apps are more popular than nutrition apps, with 3 times as many fitness app than nutrition app users. For example, in a representative survey in Germany, 17% reported to use an mHealth app, of which 67% were using a fitness app and 39% a nutrition app [22]. In addition, fitness apps were mostly used several times a week, whereas nutrition apps were typically used on a daily basis. This mirrors the actual frequency of the behavior, as fitness apps are used to track specific activities such as running or working out [74], whereas nutrition apps often require that all meals are logged to provide meaningful measures and feedback. Hence, one obvious reason for the marked difference in usage of nutrition and fitness apps might be that physical activity often is tracked automatically by using smartphone sensors [75] or wearables [22,76], whereas food intake has to be tracked manually. Manual entries in food journals can be effortful and time-consuming [77,78], and therefore, fewer people might be willing to monitor their diet. Some attempts have been made to reduce effort in food journaling, for example, by including barcode scanners, digital scales [79], or reducing extensive food databases to a list of food groups [80], but these features have yet to be included in commercially available nutrition apps.

### Stages of Behavioral Adoption

By using a stage model approach, this study expanded the dichotomy of mHealth app users and nonusers and shed more light on the psychological differences between nonacting participants. In the behavior adoption process, it is assumed that people move from a state of being unaware but starting to form opinions (stage 1) to a decision-making stage where they become engaged. They may decide to adopt the behavior (stage 2) or decide not to take action (stage 3). In this study, the two behavioral domains differed particularly in respect to the prevalence of stage 1 (“unengaged”) as half of the participants stated that they had never thought about using a nutrition app and less than one-third stated they had never thought about using a fitness app. In comparison, similar prevalence rates for stages 2 (“decided to act”) and 3 (“decided not to act”) emerged for nutrition and fitness apps. Previous research has shown that people who have not yet decided often show different responses to information and are often less resistant to persuasion than people who have reached a definite position on an issue, even if they have not yet acted on their opinions [43]. Accordingly, there seems to be greater potential to increase a nutrition app uptake using tailored information to foster the transition from being “unengaged” to becoming engaged, for example, by promoting apps that target the potential user’s health needs during medical counseling. These results also underline the importance of developing quality criteria and guidance for consumers and medical personnel to decide which apps to use or recommend [56].

A substantial number of participants stated that they had “decided not to act” (stage 3), which poses a qualitatively different transition barrier and therefore requires a different approach to changing beliefs and attitudes than for people in stages 1 or 2. A wealth of psychological research shows that people have a tendency to adhere to their own beliefs, which is challenging to overcome. In this case, providing information, for example, about the pros and cons of the target behavior, which has been effective for supporting people in the early stages of the behavioral adoption process [43], might be less effective. Transition might be more likely to be motivated by social influences such as significant others or social norms [34,81,82]. One might even argue that it is too costly to target this group and therefore more effective to focus on other groups of nonusers.

Although this study recorded few nonusers who had “decided to act” (stage 2), this group represents a qualitatively different and important target group for interventions. A great body of research suggests (1) that there are important gaps between intending to act and carrying out this intention, and (2) that helping people develop specific implementation plans that spell out the when, where, and how of goal striving in advance can reduce these barriers [83,84]. Such detailed implementation information is however seldom effective for people in stages 1 (“unengaged”) or 3 (“decided not to act”). Likewise, perceived self-efficacy seems particularly important for the transition from “decided to act” to taking action (eg, [34,85,86]).

Participants in the “acting” stage (stage 4) showed a significant different pattern of a preference for a deliberative or an intuitive style when making eating-related decisions. As expected, the current nutrition app users showed higher preference for deliberation than intuition, whereas “unengaged” nonusers (stage 1) showed a greater preference for intuition than deliberation. Accordingly, nutrition apps seem to be especially appealing to people who tend to decide what to eat after conscious reflection. mHealth apps are targeted toward this deliberative decision-making style by helping to gain insight into and control over energy intake, for example, by allowing self-monitoring and providing instruction [52]. Interestingly, participants in stage 2 (“decided to act”) expressed interest in using nutrition apps, although reporting a lower preference for deliberation and a higher preference for intuition than the current app users. This might indicate that the mismatch between the design of current available apps and preferred decision-making styles creates a significant transition barrier. Developing apps that are more tailored to an intuitive decision-making style might motivate higher stage transition rates. For example, this might be achieved by associating health behaviors with positive emotions (eg, [87]) or including game-like features, which might also increase the likelihood of habit formation [88]. However, it has yet to be investigated which app features and behavior change techniques [89] best support an intuitive decision-making style, and whether including these features actually leads to increased mHealth app adoption. As differences in preferred decision-making style between fitness app adoption stages were similar but less pronounced than differences between nutrition app adoption stages, results highlight that psychological correlates of mHealth app use are behavior-specific and therefore need to be investigated separately for different health behaviors (cf, [90]). Moreover, it is important to note that preferred decision-making style was only assessed for eating-related decisions. Thus, future studies need to test for further differences between fitness app adoption stages and the preferred decision-making style for physical activity.

In line with previous research [5], participants in the “acting” stage (stage 4) were younger than “unengaged” nonusers (stage 1). This might be due to a general higher interest in the use of mobile technology, as indicated by a higher proportion of younger smartphone owners [91] and younger people being more convinced of the efficacy of mHealth apps [4]. Moreover, the results of this study show that current nutrition app users are less educated than “unengaged” nonusers. This is in contrast with previous studies describing mHealth app users as being more educated. One reason for this difference might be that the present sample was recruited onsite as part of a cohort study, rather than online as with most previous studies. The present sample includes a broader age range and potentially less technology savvy participants. Moreover, the continuous measure used might also have had an impact as previous studies compared participants with high school and university degrees [4,5,23]. The participants in this study were generally highly educated. Moreover, the observed differences in level of education between stages were small [92]. In contrast, no such relationships were found for fitness apps, suggesting that gender and education differences might be more pronounced for nutrition than for fitness app use.

Although no differences in psychological, behavioral, and sociodemographic variables were found between “acting” users (stage 4) and “disengaged” nonusers (stage 5), the two groups differ substantially in their mHealth app use behavior. Although one might argue that “disengaged” nonusers ceased using an app because they had reached their goal, research suggests that most “disengaged” nonusers might rather have abandoned their goal [19]. This lack of engagement could, for example, be overcome by using effective behavior change techniques that help maintain the intention or the behavior [93], for example, by boosting self-efficacy or prompting planning [38]. Moreover, users might disengage from the app because tracking is too time-consuming or not interesting enough in the long term [5]. Developments in mobile technologies such as image-based assessment methods for dietary intake [94] hold great promise for reducing user burden, which might in turn boost user motivation. Thus, when further developing and testing the stage model presented in this study, models of engagement with digital behavior change interventions can provide valuable insights as they have already identified many potential transition barriers and enablers for the transition from “acting” to “disengagement” (cf, [95]). Furthermore, engagement models might also provide further insights into transition barriers as well as enablers for the transition to the “acting” stage and re-engagement [96].

In line with previous research [4,24,25], no significant differences between stages of adopting nutrition apps were found with respect to a healthy eating style and BMI, and differences found between stages of adopting fitness apps were small [92]. This might be explained by the various reasons for using mHealth apps: Although some people use them to lose weight [19], others use them without any intention to change their behavior, for example, to maintain their weight [97] or to learn more about their physical activity or eating patterns [77]. However, to examine the effect on actual changes in dietary patterns or related outcome such as BMI, longitudinal studies such as randomized control trials are needed. Although there has been much enthusiasm for delivering interventions through mobile devices such as smartphone apps, academic research on the development and evaluation of these mobile devices is at an early stage. Most currently available devices and programs have not been empirically evaluated, and the existing studies have predominantly focused on clinical samples, including text message–based mobile interventions [98-102]. Recently, Schoeppe et al [103] identified 27 studies in 6926 publications from 2006 to 2016 that used a smartphone app to improve diet and/or physical activity as a health precaution with mixed results: only 7 of the 13 studies targeting diet and 14 of the 21 targeting physical activity reported significant improvement. As most current mHealth apps focus more on user interface aspects to keep consumers engaged than evidence-based behavior change methods [104,105], incorporating effective behavior change techniques [89,106,107] might be a promising avenue for further research.

### Limitations

A strength of the study is the large sample, which represents a wide age range and was recruited onsite from the community. Although mean BMI and age were comparable to the general German population, females were overrepresented and both the university entrance diploma and the university degree rate were above the national average, potentially limiting the generalizability of the findings. Furthermore, the study was advertised as a health check; thus, the participants might have been more interested in their health than the average citizen, possibly boosting mHealth app use rates.

### Conclusions

Still, the mHealth app usage rates found both in this study and in previous research (eg, [6,22]) were low, underlining the potential to engage more people in the use of mHealth apps. Using a behavior stage model approach to describe the process of adopting mHealth apps revealed motivational stage differences between nonusers, including being “unengaged,” “decided not to act,” “decided to act,” and being “disengaged,” which might contribute to a better understanding of the process of adopting behavior changes and tailoring interventions to foster transitions between stages.

## Appendix

Multimedia Appendix 1 Generation of adaptations of the PAPM (Weinstein, & Sandman, 1992) and Preference for Intuition and Deliberation (Betsch, 2004).

Multimedia Appendix 2 Descriptive statistics of sociodemographic and behavioral correlates of fitness app adoption.

## Abbreviations

BMI: body mass index
CFI: comparative fit index
E-PD: E-PID subscale preference for deliberation
E-PI: E-PID subscale preference for intuition
E-PID: Preference for Intuition and Deliberation in Eating Decision-Making
HAPA: health action process approach
PAPM: precaution adoption process model
RMSEA: root mean square error of approximation
SRMR: standard root mean square residual
TTM: transtheoretical model of health behavior change
