# Supplementary material for: Describing the Process of Adopting Nutrition and Fitness Apps: Behavior Stage Model Approach
Source: JMIR Mhealth Uhealth. 2018 Mar 13;6(3):e55. doi: 10.2196/mhealth.8261 (PMC5871740; doi:10.2196/mhealth.8261)
Supplement: Multimedia Appendix 1 [file mhealth_v6i3e55_app1.pdf]

Supplementary File: Measures

Measure used in the current study	Original measure	Adaptation	Items (translated)
Stage model for the adoption process of mHealth apps (nutrition and fitness)	Precaution Adoption Process Model (Weinstein & Sandmann, 1992)	The adaptation is based on the Stage Model of Health-related Awareness and Action (*Stufenmodell gesundheitlichen Bewusstseins und Handelns*, Renner & Hahn, 1996) that consists of six stages:<br>- Stage 0: ‘unaware of issue’<br>- Stage 1: ‘unengaged by issue’<br>- Stage 2: ‘deciding about acting’<br>- Stage 3a: ‘decided to act’/ Stage 3b: ‘preparation’/ Stage 3c: ‘decided not to act’<br>- Stage 4: ‘acting’<br>- Stage 5: ‘break off acting’<br><br>As only participants were included who owned a mobile device, it was very unlikely that these participants did not know mhealth apps at all. Hence, we decided to drop Stage 0. As downloading an app is a simple process involving little or no costs as well as little preparatory effort and to make the model economical, we decided to drop Stages 2 and 3b. Stage 5 was renamed	*If you own a smartphone or tablet:*<br>Do you think about using an app to track your fitness/ nutrition? *Please choose one option per column.*<br>(1) I have never thought about using an app for that. *(Stage 1)*<br>(2) I have thought about using an app for that, but so far I did not do it. *(Stage 2)*<br>(3) I have thought about

Measure used in the current study	Original measure	Adaptation	Items (translated)
		‘disengaged’ to take on common terminology in accordance with the literature on engagement with smartphone apps.	using an app for that, but it is not necessary for me to do it. *(Stage 3)*<br>(4) I am currently using an app for that and intend to continue to use it. *(Stage 4)*<br>(5) I have used an app for that, but I do not use it anymore. *(Stage 5)*
Preference for Intuition and Deliberation in Eating ecision-making (E-PID; König et al., in prep.)	Preference for Intuition and Deliberation (Betsch, 2004)	The original measure consists of 19 items that measure a general preference for intuition and deliberation when making decisions. As Parchur & Spaar (2015) noted, these preferences may differ between domains. Therefore, we developed an concise measure for eating-related decision making preferences by rephrasing the	(1) When deciding what to eat, I rely on my gut feeling.[a]<br>(2) With most eating decisions, it makes sense to completely

Measure used in the current study	Original measure	Adaptation	Items (translated)
		seven items that were directly transferable to eating-related decisions. For the E-PI subscale, these were items no. 4, 12 and 18 from the original questionnaire. For the E-PD subscale, items no. 1, 5, 7 and 16 were adapted (c.f. Betsch, 2004).	rely on your feelings. [a]<br>(3) I am an intuitive eater. [a]<br>(4) Before I make eating decisions, I usually think about it. [b]<br>(5) I think more about my plans and goals relating to my eating behavior than other people. [b]<br>(6) I prefer making plans about my eating behavior instead of leaving it to chance. [b]<br>(7) I reflect on my eating behavior. [b]<br>Scale: (1) I do not agree to (5) I agree

Note. [a] E-PI, [b] E-PD

References

Betsch C. Präferenz für intuition und deliberation (PID). *Zeitschrift für Differentielle und Diagnostische Psychologie.* 2004;25(4):179-197. DOI: 10.1024/0170-1789.25.4.179

König LM, Sproesser G, Schupp HT, Renner B. Preference for Intuition and Deliberation in Eating Decision-making: Scale validation and association with health. Manuscript in preparation.

Pachur T, Spaar M. Domain-specific preferences for intuition and deliberation in decision making. *Journal of Applied Research in Memory and Cognition.* 2015;4(3):303-311. DOI: 10.1016/j.jarmac.2015.07.006

Renner B, Hahn A. Prozeßmodelle zum Gesundheitsverhalten [Process models of health behavior]. In: Renner B, Hahn A, Schwarzer R, eds. *Risiko und Gesundheitsverhalten: Deokumentation der Meßinstrumente des Forschungsprojekts "Berlin Risk Appraisal and Health Motivation Study" (BRAHMS)*. Berlin: Freie Universität Berlin; 1996.

Weinstein ND, Sandman PM. A model of the precaution adoption process: evidence from home radon testing. *Health Psychology.* 1992;11(3):170-180. DOI: 10.1037/0278-6133.11.3.170
