# Supplementary material for: Describing the Process of Adopting Nutrition and Fitness Apps: Behavior Stage Model Approach
Source: JMIR Mhealth Uhealth. 2018 Mar 13;6(3):e55. doi: 10.2196/mhealth.8261 (PMC5871740; doi:10.2196/mhealth.8261)
Supplement: Multimedia Appendix 2 [file mhealth_v6i3e55_app2.pdf]

Supplementary File: Results

Table 1

Descriptive statistics of sociodemographic and behavioral correlates of fitness app adoption.

	Gender[1]		Age	Years of education	BMI	Healthy eating style
	Female	Male	*M* (*SD*)	*M* (*SD*)	*M* (*SD*)	*M* (*SD*)
Stage 1 ‘unengaged’	253 (1.1)	130 (-1.1)	47.18 (17.00)	15.86 (2.39)	24.15 (3.46)	4.47 (0.93)
Stage 2 ‘decided to act’	69 (1.4)	29 (-1.4)	37.34 (15.68)	15.60 (2.52)	23.62 (3.11)	4.20 (0.88)
Stage 3 ‘decided not to act’	155 (0.9)	78 (-0.9)	37.51 (16.28)	16.21 (2.39)	23.93 (3.93)	4.30 (0.88)
Stage 4 ‘acting’	151 (-2.8)	115 (2.8)	34.96 (14.12)	15.73 (2.40)	24.27 (3.41)	4.23 (0.85)
Stage 5 ‘disengaged’	101 (-0.3)	60 (0.3)	34.00 (13.68)	15.74 (2.39)	24.18 (3.99)	4.25 (0.84)

Note. [1] For gender, the number of participants in the cell and the standardised adjusted residuals (in brackets) are displayed.

Table 2

Descriptive statistics of psychological correlates of fitness app adoption.

	Preference for intuition	Preference for deliberation
	M (*SD*)	*M* (*SD*)
Stage 1 ‘unengaged’	3.41 (0.84)	3.00 (0.98)
Stage 2 ‘decided to act’	3.32 (0.85)	3.32 (0.95)
Stage 3 ‘decided not to act’	3.29 (0.80)	3.28 (0.92)
Stage 4 ‘acting’	3.18 (0.85)	3.36 (0.96)
Stage 5 ‘disengaged’	3.41 (0.80)	3.19 (0.92)
